# Dengue fever with diffuse cerebral hemorrhages, subdural hematoma and cranial diabetes insipidus

**DOI:** 10.1186/s13104-016-2068-5

**Published:** 2016-05-10

**Authors:** Nayomi Shermila Jayasinghe, Eranga Thalagala, Milanka Wattegama, Kanapathipillai Thirumavalavan

**Affiliations:** General Medical Unit, National Hospital of Sri Lanka, Colombo, Western Province Sri Lanka

**Keywords:** Dengue fever, Intracranial haemorrhage, Subarachnoid haemorrhage, Subdural hematoma, Cranial diabetes insipidus, Case report

## Abstract

**Background:**

Neurological manifestations in dengue fever occur in <1 % of the patients and known to be due to multisystem dysfunction secondary to vascular leakage. Occurrence of wide spread cerebral haemorrhages with subdural hematoma during the leakage phase without profound thrombocytopenia and occurrence of cranial diabetes insipidus are extremely rare and had not been reported in published literature earlier, thus we report the first case.

**Case presentation:**

A 24 year old previously healthy lady was admitted on third day of fever with thrombocytopenia. Critical phase started on fifth day with evidence of pleural effusion and moderate ascites. Thirty one hours into critical phase she developed headache, altered level of consciousness, limb rigidity and respiratory depression without definite seizures. Non-contrast CT brain done at tertiary care level revealed diffuse intracranial haemorrhages and sub arachnoid haemorrhages in right frontal, parietal, occipital lobes and brainstem, cerebral oedema with an acute subdural hematoma in right temporo- parietal region. Her platelet count was 40,000 at this time with signs of vascular leakage. She was intubated and ventilated with supportive care. Later on she developed features of cranial diabetes insipidus and it responded to intranasal desmopressin therapy. In spite of above measures signs of brainstem herniation developed and she succumbed to the illness on day 8. Dengue was confirmed serologically.

**Conclusions:**

Exact pathophysiological mechanism of diffuse cerebral haemorrhages without profound thrombocytopenia is not well understood. Increased awareness and high degree of clinical suspicion is needed among clinicians for timely diagnosis of this extremely rare complication of dengue fever. We postulate that immunological mechanisms may play a role in pathogenesis. However further comprehensive research and studies are needed to understand the pathophysiological mechanisms leading to this complication.

## Background

Dengue fever is caused by a flavi virus, which is a vector borne ribonucleic acid (RNA) virus [1] with four antigenically distinct serotypes (Dengue 1–4). Neurological manifestations are rare compared to other complications of the disease. Encephalopathy, Encephalitis, Aseptic meningitis, intracranial hemorrhages, thrombosis, mononeuropathies/polyneuropathies, Guillain–Barre syndrome and myelitis have been reported [2]. Neurological manifestation in dengue hemorrhagic fever usually results from multisystem dysfunction secondary to liver failure, cerebral hypo perfusion, electrolyte imbalance, shock, cerebral edema, and hemorrhage related to vascular leak [3, 4]. The occurrence of brain hemorrhage in a case with dengue shock can be serious and leads to death [5]. The occurrence of brainstem hemorrhage can be a very serious fatal situation [6]. We report this case of dengue hemorrhagic fever with multiple intracranial, sub arachnoid hemorrhages and sub-dural hematoma causing brainstem herniation with respiratory depression complicated with cranial diabetes insipidus.

## Case presentation

A 24 year old previously healthy lady was admitted to a local hospital on third day of fever with arthralgia and myalgia without any bleeding manifestations. On examination she was hemodynamically stable with a pulse rate of 80 beats per minute and blood pressure of 110/70 mmHg. There was no postural drop in blood pressure, pleural effusions or ascites to suggest vascular leakage. She did not have symptoms or signs of neurological involvement on admission. Full blood count revealed white blood count (WBC) 3.26 × 10^9^/L with differentials of Neutrophils 2.0 × 10^9^/L, Lymphocytes 1.1 × 10^9^/L and Monocytes 0.1 × 10^9^/L. Hemoglobin (Hb) 13.2 g/dL, platelet count 80 × 10^9^/L and hematocrit (Hct) 38 %.

Tentative diagnosis of dengue fever without fluid leakage was diagnosed as clinical features and investigations are characteristic of the infection. Fluid management and monitoring of vital parameters was started according to National Dengue management guidelines [7].

On fifth day of fever patient developed signs of fluid leakage. Hematocrit was 46 % which is a >20 % rise from baseline value. Other signs of leakage were right sided pleural effusion and ascites which were confirmed radiologically and low albumin levels (2.4 g/dl). Critical phase monitoring and fluid management was instituted according to the guideline. Patient did not have bleeding manifestations. Thirty one hours into critical phase she complained of severe headache and difficulty in breathing. Examination revealed a drowsy patient with increased rigidity of all four limbs and sinus tachycardia. Reflexes were exaggerated in all four limbs without focal neurological signs or papilledema. No tonic–clonic movements were noted. Blood pressure remained normal. Peripheral oxygen saturation was 92 % on air. Bilateral pupil sizes were 3 mm with slow reaction to light. Possibility of intracranial hemorrhage with brainstem involvement was suspected. Endotracheal intubation and ambu ventilation was initiated as she rapidly developed respiratory depression and deterioration of Glasgow Coma Scale (GCS) to 6/15. Hemodynamic parameters were stable with a satisfactory urine output. She was transferred to a tertiary care level for urgent brain imaging and neurosurgical opinion.

**Fig. 1:**
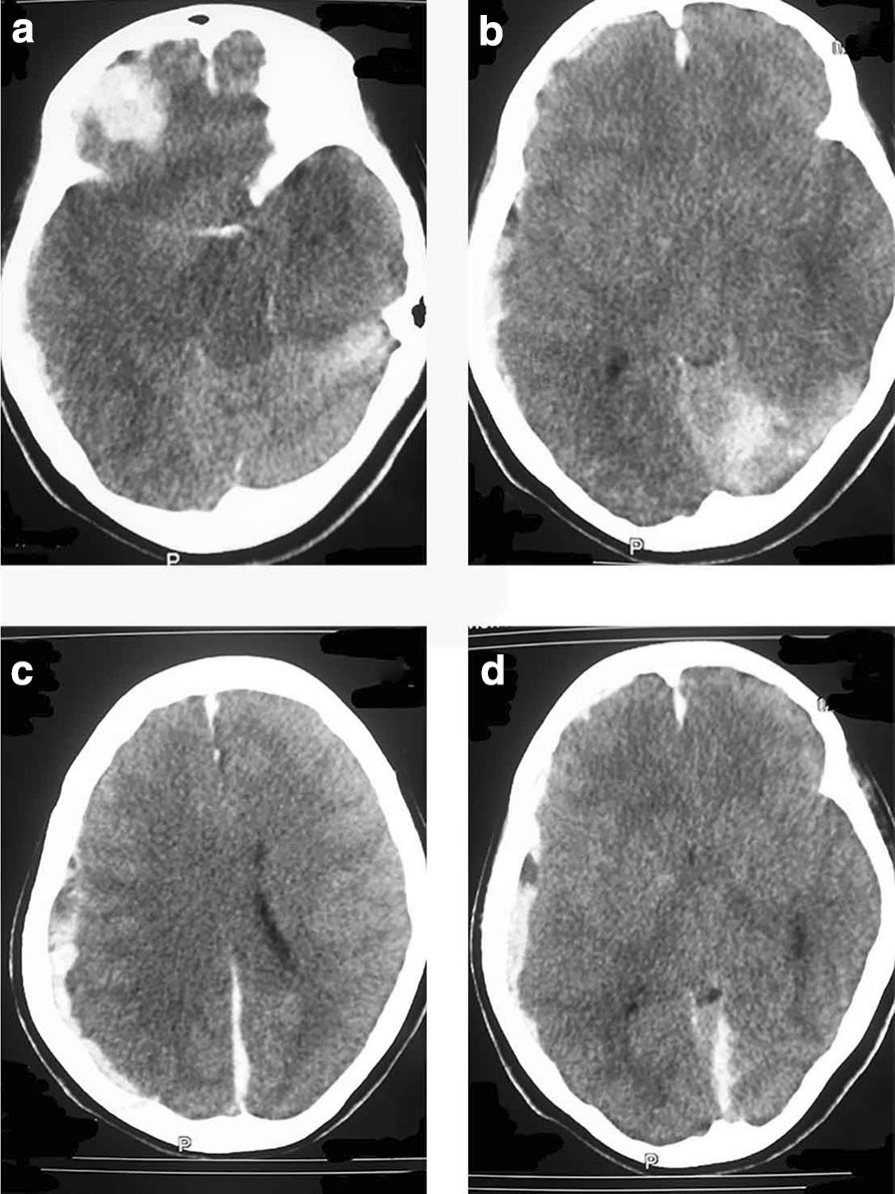
**a**, **b** Non contrast computer tomography (CT) brain showing *right* frontal, *left* parietal and occipital lobe subarachnoid hemorrhages and intra cranial hemorrhages. **c**, **d** Non contrast computer tomography (CT) brain showing *right* subdural hematoma

Urgent non contrast computer tomography (CT) of brain showed multiple sub arachnoid hemorrhages in right frontal, left parietal and occipital lobes. It also showed right sided sub dural hematoma and gross cerebral edema compressing bilateral lateral ventricles, third ventricle and brainstem. (As reported by consultant radiologist) (Fig. 1).

Urgent neurosurgical opinion was taken regarding further management and was decided to manage conservatively considering the critical phase of dengue fever with low platelet counts. Patient was immediately transferred to the intensive care unit for further management. As advised by the neurosurgical team patient was started on intravenous Dexamethasone 8 mg three times daily, Intravenous Vitamin K 10 mg daily, intravenous Tranexamic acid 500 mg three times daily, Phenytoin Sodium 100 mg two times daily and Folic acid 5 mg daily. Intravenous fluids were given according to the critical phase managing guidelines. Ventilatory support was continued.

At the time of developing cerebral hemorrhages platelet count was 40 × 10^9^/L and coagulation profile was normal. Patient did not have signs of bleeding from other sites. Alanine Transaminase level (ALT) was 312 IU/L, Aspartate transaminase (AST) was 666 IU, Serum albumin level 2.4 g/dl, serum creatinine was 49 µmol/L. Blood picture did not show any evidence of disseminated intravascular coagulation or thrombotic thrombocytopenic purpura. Due to multiple cerebral hemorrhages it was decided to transfuse six units of platelets when the platelet count was 15 × 10^9^ on seventh day of illness. *ABCS* (*A*cidosis, *B*leeding, *C*alcium and *S*ugar) were monitored and corrected. Hematocrit dropped to 29 from 46 % despite adequate fluid replacement and there was unexplained tachycardia. 5 ml/kg of packed cells were transfused following which the hematocrit normalized. Venous blood gas analysis did not show evidence of metabolic acidosis. Serum ionized calcium was 1.12 mmol/L and it was corrected with intravenous calcium gluconate. Capillary blood sugar level was within the normal range throughout the illness.

On sixth day of the illness patient developed polyuria with >500 ml of urine per hour (>12 L per day) with hypernatremia. Serum sodium level was 150 mmol/L, Serum Potassium was 3.0 mmol/L. Urine specific gravity was 1.005 and urine osmolality was 153 mOsm/kg. A diagnosis of cranial diabetes insipidus was made and treated with intra nasal Desmopressin sprays following which polyuria and hyper osmolality settled. Platelet count continued to drop even after completion of critical phase reaching a lowest value of 15 × 10^9^ on 7th day of illness.

Despite intensive medical therapy patient’s clinical condition deteriorated with appearance of signs of brainstem herniation. Pupils were fixed dilated with no reaction to light. Patient developed per vaginal bleeding and punctures site bleeding. Blood pressure gradually dropped needing ionotropic support. Patient succumbed to illness on day eighth.

## Discussion

This case highlights few important points about dengue fever. Firstly, occurrence of multiple cerebral hemorrhages while the platelet count is not very low. Secondly, the occurrence of subdural hematoma and intra cranial hemorrhages in a previously healthy lady purely secondary to dengue fever. Thirdly, occurrence of cranial diabetes insipidus in dengue fever. We aim to discuss these points in the following text.

The Dengue virus, being a member of family *flaviviridae* is well known to cause hemorrhagic manifestations. Thrombocytopenia, capillary leakage and various degrees of coagulopathy has been attributed to these bleeding manifestations. Minor bleeding manifestations, most commonly skin petechiae or bruising are apparent in many patients with dengue hemorrhagic fever (DHF). But major hemorrhage is unusual. If severe bleeding does occur, it is almost invariably in patients with profound or protracted shock who also have evidence of multiple-organ failure [8]. Cerebral hemorrhages in severe dengue shock syndrome with severe thrombocytopenia have been reported. But cases of diffuse cerebral hemorrhages during moderate thrombocytopenia were not found in literature. Occurrence of cranial diabetes insipidus following dengue infection was also not reported in published literature to our knowledge (search in PubMed with keywords ‘dengue’ and ‘diabetes insipidus’ in any field).

On admission patient had classic history and investigations to suggest uncomplicated dengue fever without fluid leakage and was managed accordingly in par with National Dengue Guidelines with close monitoring. Dengue infection was confirmed by positive Dengue IgM serology. Fluid leakage phase was evidenced by development of pleural effusion and ascites and was supported biochemically with >20 % rise in hematocrit and low serum albumin (2.4 g/dL). Pleural effusion and ascites was confirmed by ultrasound scan. Development of sudden headache with respiratory depression and low Glasgow Coma Scale raised the suspicion of intracranial hemorrhage with brainstem involvement and it was confirmed by brain imaging. Cerebral vessel angiogram did not reveal any other causes of intra cranial hemorrhages like leaking/ruptured aneurysms. Occurrence of diffuse cerebral hemorrhages when platelet count was 40 × 10^9^/L without multi organ failure or disseminated intravascular coagulation was an atypical presentation. High index of clinical suspicion lead to early diagnosis and initiation of relevant management. Instituting a neurosurgical procedure at the height of dengue critical phase with thrombocytopenia in a patient with brainstem compression was a challenging decision to make. Considering the risks versus benefit conservative management was decided rather than invasive surgery. Measures to reduce cerebral edema were initiated along with fluid management for critical phase. Occurrence of acute subdural hematoma in dengue fever is rarely reported [9]. Mechanisms leading to this phenomenon are poorly understood. Six units of platelets were transfused as she had evidence of acute intracranial bleeding even though the platelet count was not very low. Development of active bleeding with moderate thrombocytopenia and normal clotting profile may be explained by platelet functional defects which are known to occur in dengue infection [10]. However, we did not have adequate facilities to carry out platelet function tests.

Diabetes insipidus was diagnosed according to the diagnostic criteria of polyuria (>12 L of urine/day), hypernatremia (Serum sodium level 150 mmol/L) and production of diluted urine (Urine specific gravity 1.005 and urine osmolality 153 mOsm/kg). Nephrogenic diabetes insipidus was unlikely considering the fact that the renal functions were normal and absence of any drugs or illnesses causing nephrogenic diabetes insipidus. Cranial diabetes insipidus was more likely considering significant cerebral hemorrhages and edema. Performing the water deprivation test as the confirmatory test was not carried out as she needed strict fluid management for dengue leaking phase. Correction of polyuria, serum and urine osmolality changes in response to desmopressin nasal sprays was retrospectively taken as confirmation of cranial diabetes insipidus. Occurrence of diabetes insipidus as a direct effect of dengue virus on the hypothalamus or as a secondary effect due to cerebral hemorrhages was a point of interest to look into.

## Conclusions

This case elaborates some rare and life threatening complications of a common infection and it describes occurrence of cranial diabetes insipidus in dengue fever for the first time. It is important to have a high degree of clinical suspicion when a patient presents with atypical symptoms, to diagnose rare complications. Prompt diagnosis of such complications can alter the clinical course as well as the outcome of the disease. Further research should be encouraged with regards to pathophysiological mechanisms of dengue infection leading to central nervous system involvement.

## Abbreviations

RNA: ribonucleic acid
WBC: white blood count
Hb: hemoglobin
Hct: hematocrit
GCS: Glasgow coma scale
CT: computer tomography
ALT: alanine transaminase level
AST: aspartate transaminase
ABCS: acidosis, bleeding, calcium and sugar
DHF: dengue hemorrhagic fever
